# Loading and activation of DNA replicative helicases: the key step of initiation of DNA replication

**DOI:** 10.1111/gtc.12040

**Published:** 2013-03-05

**Authors:** Yan Li, Hiroyuki Araki

**Affiliations:** Division of Microbial Genetics, National Institute of GeneticsYata 1111, Mishima City, Shizuoka, 411-8540, Japan

## Abstract

Evolution has led to diversification of all living organisms from a common ancestor. Consequently, all living organisms use a common method to duplicate their genetic information and thus pass on their inherited traits to their offspring. To duplicate chromosomal DNA, double-stranded DNA must first be unwound by helicase, which is loaded to replication origins and activated during the DNA replication initiation step. In this review, we discuss the common features of, and differences in, replicative helicases between prokaryotes and eukaryotes.

## Introduction

All organisms must copy their genetic information faithfully to their offspring. The copy process, called chromosomal DNA replication, is very sophisticated and is regulated strictly within every single cell. Differences in cell structures and organization of genomic DNA between prokaryotic and eukaryotic cells suggest that their DNA replication processes must also exhibit differences, although more mechanisms that are common may also be shared.

**Figure 1:**
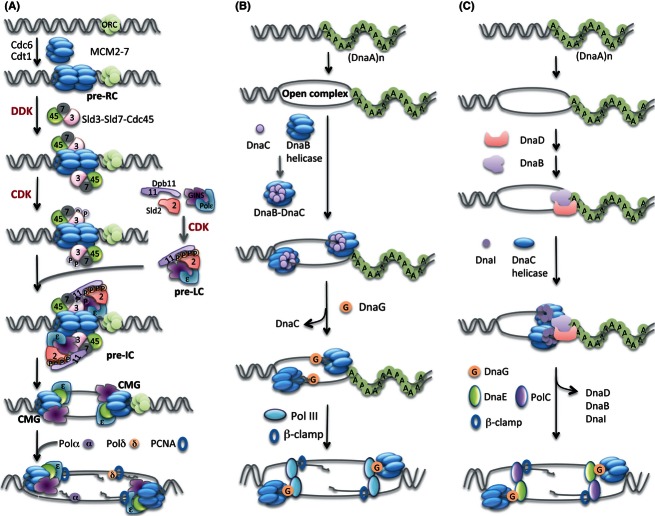
Origin loading and activation of replicative helicases. (A) In eukaryotes, the first step is loading of Mcm2–7 helicase onto the replication origin region that depends on origin recognition heterohexamer ORC and two loading proteins Cdc6 and Cdt1. In budding yeast *S. cerevisiae*, the subsequent activation of helicase requires DDK and CDK, recruiting other replication initiation proteins including a Sld3-Sld7-Cdc45 complex and then a preloading complex (pre-LC) composed of Sld2, Dpb11, GINS tetramer and polymerase DNA Pol ε onto the head-to-head double MCM hexamers to form a huge pre-initiation complex (pre-IC) at origin regions, immediately followed by remodeling that leads to the activation of CMG helicase. This process probably switches MCM from encircling dsDNA to ssDNA. Next, the active CMG works together with DNA polymerases, Pol α, Pol δ and Pol ε to duplicate DNA. (B) In Gram-negative *E. coli*, the initiator DnaA recognizes the bacterial replication origin *oriC* and melts a specific origin sequence to form an open complex in the presence of ATP. Then, DnaB–DnaC complex is loaded on each separated single-stranded DNA. The binding of primase DnaG with DnaB helicase and the primer synthesis induce removal of DnaC from DnaB. The DnaC dissociation requires ATPase activity. Subsequent loading of DNA replicative polymerase Pol III and β-clamp triggers replication elongation. (C) In Gram-positive *B. subtilis*, DnaA binds and melts *oriC* sequence, followed by loading of DnaD and DnaB orderly onto the origin region. DnaC helicase and DnaI assemble to the origin subsequently in the presence of ATP. The remodeling including removal of DnaD, DnaB and DnaI and the assembly of primase DnaG, two DNA polymerases, DnaE and PolC, and β-clamp activates helicase and replication fork movement.

Chromosomal DNA replication initiates from origins in a chromosome that an initiator specifically recognizes. Other replisome components are recruited to the initiator-associated origins in a coordinated manner. The latter step includes loading of the replicative DNA helicases onto the origin region. DNA helicases are enzymes required for both DNA replication initiation and elongation steps, where they catalyze the unwinding of duplex DNA strands and translocate along the bound DNA strands using the energy provided by ATP hydrolysis, thereby providing DNA templates for DNA polymerases to synthesize new strands (Fig. 1). The key role of helicases makes their loading to the origin and the subsequent activation by other regulatory factors one of the most crucial events in the replication initiation step.

In this review, we focus on the initiation step of DNA replication, especially loading of replicative DNA helicase onto origins and discuss its diversity as well as general features in various organisms.

## Helicase unwinds double-stranded DNA for DNA polymerase to synthesize DNA

Primase synthesizes primer RNA, and DNA polymerases extend the DNA strand from the primer on a single-stranded template DNA. To form single-stranded DNA, helicase uses the energy from ATP hydrolysis to break the hydrogen bonds that connect two single strands together in duplex oligonucleotides. The replicative helicases specialize in DNA replication events where they unwind the double helix to provide single strands as the templates for primase and DNA polymerase to synthesize new DNA strands. Replicative helicases are ubiquitous, evolutionarily conserved proteins. Their loading and activation at chromosomal replication origins are the most important events in replication initiation. The replicative helicases perform activities such as binding to DNA strands, ATP-binding and ATP hydrolysis activities, double-strand unwinding and translocation in a specified direction coupled with ATP hydrolysis. Although there are some sequence differences, the unwinding activity of the helicase catalytic core is not sequence specific.

**Figure 2:**
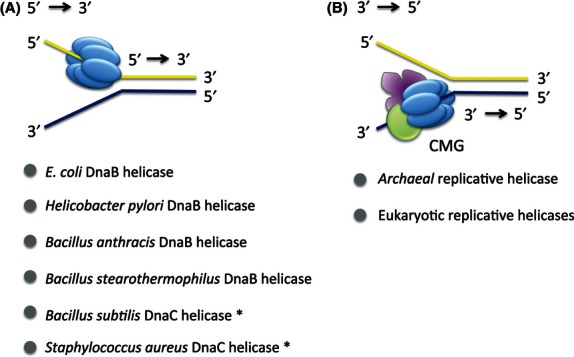
Unwinding polarity of replicative helicases. (A) Gram-negative bacteria *E. coli* and *Helicobacter pylori* helicase hexamer unwind double-stranded DNA in the direction of 5′ to 3′. Similarly, Gram-positive bacteria *Bacillus anthracis* and *Bacillus stearothermophilus* also showed a 5′ to 3′ unwinding activity. The DnaC helicase of *Bacillus subtilis* with 45% and 82% identical amino acid sequences to the counterpart in *E. coli* and *Bacillus stearothermophilus,* respectively, is speculated to have the same 5′ to 3′ helicase activity. Gram-positive *Staphylococcus aureus* DnaC helicase has 44% and 58% homology with *E. coli* DnaB and *B. subtilis* DnaC replicative DNA helicase, respectively, suggesting the same unwinding polarity as 5′ to 3′. (B) Both archaeal and eukaryotic CMG helicase complexes unwind double-stranded DNA in the direction 3′ to 5′.

The most characterized replicative DNA helicase is the *Escherichia coli* DnaB homohexamer. The N-terminal domain of the DnaB protein acts in primase binding, and the C-terminal domain with Walker A and B motifs, and arginine finger inside engages with ATP and associates with the DnaC helicase loader (Yu *et al*. 1996; Lo *et al*. 2009; Makowska-Grzyska & Kaguni 2010). The DnaB hexamer translocates along the lagging-strand template in the 5′ to 3′ direction (Fig. 2). All prokaryotic cells examined so far have DnaB-like helicases. For example, *Bacillus subtilis* DnaC is an orthologue of *E. coli* DnaB helicase and also forms a homohexamer in the replisome (Table 1) (Velten *et al*. 2003).

**Table 1:** Comparative guide for replication proteins between prokaryotes and eukaryotes

	Prokaryotes			
			
Functions	*E. coli* (Gram-negative)	*B. subtilis* (Gram-positive)	Archaea	Eukaryotes
Origin recognition	DnaA⋆	DnaA⋆	Orc1⋆/Cdc6⋆	ORC⋆
Helicase loading	DnaA⋆, DnaC⋆	DnaA⋆, DnaD, DnaB, DnaI⋆	Orc1⋆/Cdc6⋆	ORC⋆, Cdc6⋆, Cdt1
Helicase	DnaB_6_	DnaC_6_	MCM⋆_6_	MCM2–7⋆
Helicase activity auxiliary factors			RecJ like protein, GINS	Cdc45, GINS
Primase	DnaG	DnaG	Primase	Primase/Pol α
Polymerases	Pol III	PolC, DnaE_BS_	Pol-B, Pol-D	Pol δ, Pol ε
Other essential proteins involved in helicase activation		DnaB, DnaD		Sld2, Sld3, Dpb11
Initiation regulation proteins				CDK, DDK,

⋆ AAA^+^ protein.

In archaea and eukaryotes, MCM acts as a helicase in DNA replication (Table 1). MCM was identified originally in yeast when screening for the genes that regulate cell cycle progression, plasmid maintenance and chromosome segregation (Bochman & Schwacha 2009). Archaea has only one MCM subunit that acts as a homohexamer. (although some archaea have multiple MCM species, only one of them works in DNA replication (Ishino *et al*. 2011; Pan *et al*. 2011)). Eukaryotes use six distinct subunits that have the MCM motif, forming the heterohexamer MCM2–7 (Bochman & Schwacha 2009). Although MCM2–7 shows a weak helicase activity (Bochman & Schwacha 2008), it displays robust activity when it forms a Cdc45–MCM2–7–GINS (CMG) complex with Cdc45 and tetraheteromeric Go–Ichi–Nii–San complex (GINS) (see below) (Moyer *et al*. 2006; Ilves *et al*. 2010). Interestingly, the CMG complex translocates along the leading-strand template in the 3′–5′ direction, opposite to the prokaryotic helicase DnaB (Fig. 2). The archaeal MCM displays helicase activity and translocates in the 3′–5′ direction, as does eukaryotic MCM2–7 (Barry & Bell 2006).

## Initiator proteins specify the chromosome loci for helicase loading

Replicative helicase is loaded onto replication origins at the initiation of DNA replication. The initiator proteins, DnaA protein in prokaryotes and ORC in archaea and eukaryotes, specify replication origins. DnaA and ORC complexes are highly conserved in eubacteria species and in archaea and eukaryotes, respectively (Table 1). Both bind to ATP/ADP, and the ATP-bound form is active during initiation.

Bacterial replication initiates from a single origin, *oriC*. *E. coli oriC* spans the minimal functional 245-bp sequence comprising two parts, the AT-rich DNA unwinding element (DUE) and the DnaA-binding region (Bramhill & Kornberg 1988). The latter contains multiple DnaA-binding sites (DnaA boxes) with various affinities; three of them bind to DnaA with high affinity through most of the cell cycle irrespective of the nucleotide form of DnaA (Cassler *et al*. 1995; Ryan *et al*. 2004), whereas the other shows relatively weak affinity for DnaA but is bound preferentially by ATP–DnaA at initiation (McGarry *et al*. 2004; Kawakami *et al*. 2005). The cooperative binding of ATP–DnaA to *oriC* forms a homomultimeric complex of 10–20 molecules (Katayama *et al*. 2010), which then leads directly to melting of DUE to generate a single-stranded DNA region (the open complex) (Fig. 1B). Several factors affect the melting. The DiaA tetramer stimulates multimerization of DnaA and consequently facilitates the melting of *oriC* DNA (Katayama *et al*. 2010). *OriC* binding to two histone-like proteins, IHF and HU, enhances the unwinding of *oriC*, whereas the other protein, Fis, inhibits it (Ryan *et al*. 2004; Chodavarapu *et al*. 2008). The unwound DNA strands provide helicase for the scaffold for the subsequent assembly onto the origin. In the following initiation events, *E. coli* DnaB helicase is loaded, by interaction with DnaA initiator, to each strand of unwound origin DNA in the complex with DnaC protein (Fig. 1B) (Katayama *et al*. 2010; Kaguni 2011).

*Escherichia coli* DnaA (amino acids (aa) 1–467) has four domains: I (aa 1–86), II (aa 87–134), III (aa 135–373) and IV (aa 374–467) (Ozaki & Katayama 2009; Katayama *et al*. 2010). Domain I bears DnaA oligomerization activity and binding activities to the helicase DnaB, HU, the initiation stimulator DiaA and some other proteins. The interaction between this domain and DnaB helicase is crucial for the loading of DnaB onto the unwound single-stranded DNA at the replication origin, *oriC* region, in the presence of DnaC (see below) (Masai *et al*. 1990; Masai & Arai 1995; Carr & Kaguni 2002). Domain II serves as an unstructured linker to connect domain I with III. Domain III contains the AAA^+^ (ATPases associated with diverse cellular activities) ATPase sequence (Iyer *et al*. 2004). As a member of the AAA^+^ ATPase family, DnaA domain III bears common features such as ATP binding and ATP hydrolysis, and oligomerization. DnaA transforms itself between the active ATP–DnaA and the inert ADP–DnaA forms at the start of replication initiation (Sekimizu *et al*. 1987; Kurokawa *et al*. 1999). Its innate ATPase activity is very weak and is DNA dependent (Sekimizu *et al*. 1987; Messer *et al*. 1999; Felczak & Kaguni 2004). Domain IV is the site that binds specifically to DnaA boxes at *oriC* (Messer *et al*. 1999; Weigel *et al*. 1999; Blaesing *et al*. 2000; Felczak & Kaguni 2004; Kawakami & Katayama 2010; Kaguni 2011). Other prokaryotes, such as *Bacillus* and *Staphylococcus aureus*, have DnaA that seems to function like that of *E. coli*, although its ATPase activity and its affinity for ATP and ADP differ slightly from those of *E. coli* (Table 1) (Kurokawa *et al*. 2009; Bonilla & Grossman 2012).

Eukaryotes start DNA synthesis from multiple origins. The ORC is the counterpart of bacterial DnaA in eukaryotes (Table 1). It was first purified from the budding yeast *Saccharomyces cerevisiae* (Bell & Stillman 1992), and its orthologues have been found in a wide range of eukaryotic species (Bochman & Schwacha 2009). This heterohexamer comprises six tightly associated subunits called Orc1, Orc2, Orc3, Orc4, Orc5 and Orc6. The complex binds to the origin sequence called autonomously replicating sequence (ARS) in chromosomes of budding yeast. One of the subunits, Orc6, is dispensable for ARS binding in budding yeast. Interestingly, Orc6 is required for both Cdt1 loading onto the origin (Chen *et al*. 2007) and for entry into S phase after pre-RC formation *in vivo* (see below) (Semple *et al*. 2006). By contrast, Orc1 to Orc5 has one or two winged-helix (WH) domains that are involved in DNA association. In addition, Orc1, Orc4 and Orc5 possess one AAA^+^ ATPase motif, whereas Orc2 and Orc3 are presumed to possess one AAA^+^-like fold each. Therefore, ORC function is tightly regulated by ATP binding and hydrolysis. A study of budding yeast showed that double-stranded origin DNA stabilizes ATP binding and inhibits ATP hydrolysis of the ORC; by contrast, any single-stranded DNA stimulates ATPase activity (Lee *et al*. 2000). Instead of recognizing specific sequences, the ORC in most metazoa may recognize specific DNA structures, such as chromatin, which can deform to fit into the binding of the ORC (Gaudier *et al*. 2007). However, unlike bacterial DnaA, the ORC alone does not unwind replication origins.

Archaea initiates DNA replication from one or several origins, depending on the species. Archaea have initiator proteins similar to ORC subunits, although the initiator protein comprises fewer molecular species, that is, from one to three (Table 1) (Barry & Bell 2006). Archaeal ORC cannot unwind origin DNA, like the eukaryotic ORC, but underwinds double-stranded origin DNA (Dueber *et al*. 2007; Gaudier *et al*. 2007).

## Helicase loader with the initiator proteins recruit helicase to replication origins

Helicase is loaded to the execution point when it works. At the initiation step of DNA replication, helicase is loaded to origins with the aid of specific factors and initiator proteins. This step requires at least one ATPase.

The DnaC protein of *E. coli* was first characterized as a helicase loader. DnaC comprises 245 aa with Walker A, Walker B and Box VII motifs, indicating itself a member of AAA^+^ ATPase family. The structural analysis of *Aquifex aeolicus* DnaC suggested that DnaC is a paralogue of DnaA. The ATP-binding and ATP hydrolysis activities play key roles, respectively, in the loading of the replicative helicase DnaB to *oriC* and its release from loaded DnaB (Fig. 1B). The full-length DnaC protein can bind to DnaB without intervention by ATP, but ATP is indispensable for DnaC to load DnaB onto the origin DNA (Davey *et al*. 2002; Davey & O'Donnell 2003). This is probably because ATP increases the affinity of DnaC for single-stranded DNA and thus promotes the loading of DnaB to single-stranded origin DNA in cooperation with DnaA (Biswas & Biswas-Fiss 2006). Several groups reported that DnaC and DnaB form a complex in which one DnaB hexamer interacts with six DnaC monomers (Wahle *et al*. 1989; San Martin *et al*. 1998). However, a more recent study suggested that three DnaC monomers interact with one DnaB hexamer (C3B6) to act as an active form in helicase loading and activation (Makowska-Grzyska & Kaguni 2010). The C3B6 model is supported by the viral DnaB6–λP3 complex, in which λP is the analogue of DnaC in bacteriophage lambda (Mallory *et al*. 1990). A slight excess of DnaC inhibits the movement of replication forks by inhibiting DnaB helicase activity *in vitro* (Allen & Kornberg 1991) and *in vivo* (Skarstad & Wold 1995), suggesting that *E. coli* DnaC protein should release from the *oriC*-associated DnaB–DnaC complex or from a stalled replication fork to activate helicase. The interaction of DnaB with dnaG primase and primer synthesis are suggested as triggers of the release of DnaC (Makowska-Grzyska & Kaguni 2010).

Low GC content Gram-positive bacteria, such as *B. subtilis* or *S. aureus*, have the conserved proteins DnaD, DnaB (not equivalent to the *E. coli* DnaB helicase) and DnaI that have been suggested to participate in helicase loading at the replication initiation (Table 1) (Bruand *et al*. 2001, 2005; Li *et al*. 2004, 2007). The associations of these proteins with *oriC* in conditional mutant cells suggest the associations are ordered in *B. subtilis* (Smits *et al*. 2010); DnaA recognizes the origin, DnaD and DnaB_*Bs*_ associate sequentially with the origin, and finally DnaC monomers are recruited one by one in a DnaI-dependent manner to form a hexameric ring (Fig. 1C) (Velten *et al*. 2003). DnaD interacts with the origin initiator, DnaA (Ishigo-Oka *et al*. 2001; Marsin *et al*. 2001) and with DnaB_*Bs*_, which associates with the membrane (Hoshino *et al*. 1987; Rokop *et al*. 2004). DnaD and DnaB_*Bs*_ proteins possess nonspecific single-stranded and double-stranded DNA-binding ability. DnaI belongs to the AAA^+^ ATPase family and has an N-terminal domain for helicase interaction and a C-terminal domain that contains ATPase and cryptic DNA-binding activities (Ioannou *et al*. 2006). DnaI also interacts with DnaB to recruit DnaC to assemble replicative helicase. These multiple components for helicase loading may be similar to the eukaryotic components Orc6, Cdc6 and Cdt1 (see below).

In eukaryotes, Cdc6 and Cdt1 play a role in loading helicase to origins (Table 1). Cdc6 was first characterized as a DNA replication factor in *S. cerevisiae* (Hartwell 1976) and is conserved from archaea to humans (Liu *et al*. 2000). Cdc6 protein belongs to the AAA^+^ ATPase family and shares some similarities with Orc1 in the Walker A and B motifs as well as the neighboring WH domain (Capaldi & Berger 2004). This is similar to the relationships between DnaA and DnaC in *E. coli* and between DnaA and DnaI in *B. subtilis* (Table 1). The ATP binding and hydrolysis by the AAA^+^ motif of Cdc6 contributes to MCM2–7 helicase loading at the origin (Randell *et al*. 2006). Cdt1 is also conserved in eukaryotes (Hofmann & Beach 1994; Maiorano *et al*. 2000; Nishitani *et al*. 2000; Whittaker *et al*. 2000; Wohlschlegel *et al*. 2000; Devault *et al*. 2002; Tanaka & Diffley 2002). In budding yeast, Cdt1 and MCM2–7 form a complex, which is recruited to origins in a Cdc6- and Orc6-dependent manner (Chen *et al*. 2007). MCM2–7 loaded to origins exists as a head-to-head double hexamer (Fig. 1A) (Evrin *et al*. 2009; Remus *et al*. 2009). It has been proposed that Orc6 has two Cdt1-binding sites and thus recruits two Cdt1–MCM2–7 complexes simultaneously (Takara & Bell 2011). As described above, Orc6 is not required for association of ORC with origin DNA but it is required for the recruitment of helicase. This multiple factor-mediated recruitment of helicase is similar to that of low GC content Gram-positive bacteria (see above).

Although most prokaryotes and eukaryotes require ATPase loader for loading helicases to origin DNA, archaea do not have a specific loader for helicase, but instead the initiator protein alone functions as the loader (Barry & Bell 2006). Even in prokaryotes, there is an exception. The DnaB helicase from *Helicobacter pylori* forms a head-to-head double hexamer, which is similar to loaded MCM2–7 in eukaryotes. This DnaB seems to load to *ori*C in the absence of the loader because it complements both *dnaB* and *dnaC* temperature-sensitive *E. coli* mutants (Stelter *et al*. 2012). Further analyses will show how these helicases are loaded onto origins.

## Formation of active helicase

The *E. coli* helicase DnaB is loaded to single-stranded DNA origins with DnaC helicase loader and is activated by the release of DnaC once primase interacts with DnaB (Katayama *et al*. 2010; Kaguni 2011). Formation of the active helicase in eukaryotic cells is more complicated. Eukaryotic MCM is loaded to double-stranded DNA at origins and forms the pre-RC complex. Subsequent association of additional proteins with origins, which is facilitated by CDK- and DDK-dependent protein phosphorylations, forms the active helicase, the CMG complex. The CMG complex was first purified and characterized from *Drosophila* embryo extracts with ATP-dependent helicase activity (Moyer *et al*. 2006) and was also found in *Xenopus* egg extract and in budding yeast (Gambus *et al*. 2006; Pacek *et al*. 2006). Further study showed that the biochemical functions of MCM, including ATP hydrolysis, helicase activity and affinity for DNA substrates, are increased markedly upon association with both Cdc45 and GINS (Ilves *et al*. 2010). The formation of the CMG complex is intriguing. CMG is obviously the true helicase working unit that tracks along the single-stranded DNA with the leading-strand DNA polymerase, Pol ε, at the replication fork (Fu *et al*. 2011; Kang *et al*. 2012), although the catalytic activity undoubtedly originates from the AAA^+^ motif of MCM proteins.

Cdc45 is crucial for DNA replication initiation and elongation and is conserved among eukaryotes in both sequence and function. It has a weak similarity in sequence with the DHH family including inorganic pyrophosphatases and *E. coli* RecJ single-stranded DNA exonucleases (Sanchez-Pulido & Ponting 2011; Krastanova *et al*. 2012). Small-angle X-ray scattering analysis predicts that, like RecJ, human Cdc45 has a compact core with two lateral extensions. Human Cdc45 binds to single-stranded DNA as predicted but does not display exonuclease activity (Krastanova *et al*. 2012). Its function is related closely with another protein, Sld3/Treslin/Ticrr, which was characterized recently as functionally conserved in different eukaryotic organisms (Table 1) (Kumagai *et al*. 2010, 2011; Sanchez-Pulido *et al*. 2010; Sansam *et al*. 2010; Boos *et al*. 2011).

GINS was identified as a heterotetramer that is essential for replication initiation and elongation (Table 1) (Kanemaki *et al*. 2003; Kubota *et al*. 2003; Takayama *et al*. 2003). It comprises four subunits Sld5, Psf1, Psf2 and Psf3, which are well conserved among eukaryotes (Labib & Gambus 2007). Most archaea have Gins15 and Gins23, which are related to Psf1 and Sld5 and to Psf2 and Psf3, respectively. Two copies each of Gins15 and Gins23 form a tetramer, similar to eukaryotic GINS (Bell 2011). Archaeal GINS interacts directly with MCM helicase, RecJ family nuclease and primase to stimulate MCM helicase activity (Makarova *et al*. 2012), suggesting a central role in archaean DNA replication.

Eukaryotic DNA replication requires two protein kinases, DDK and CDK, both of which are activated at the G1/S boundary and harmonize DNA replication with other cell cycle events. DDK phosphorylates MCM2–7 and consequently promotes association of the Sld3–Sld7 complex and Cdc45 with the pre-RC in budding yeast (Heller *et al*. 2011; Tanaka *et al*. 2011a,b). Sld3-Sld7 and Cdc45 form a complex and associate with origins in a mutually dependent manner. The loading of the complex happens even in the G1 phase for early firing origins through the residual activity of DDK (Kamimura *et al*. 2001; Tanaka *et al*. 2011a). GINS, instead, is loaded onto origins in a CDK-dependent manner (Takayama *et al*. 2003). CDK phosphorylation of yeast Sld2 and Sld3, and their subsequent binding to another replication protein Dpb11, lead to replication initiation (Tanaka *et al*. 2007; Zegerman & Diffley 2007; Tanaka & Araki 2010). The phosphorylation-dependent interaction between Dpb11 and Sld2 promotes the formation of the preloading complex (pre-LC), which contains Pol ε, GINS, Dpb11 and CDK-phosphorylated Sld2 (Muramatsu *et al*. 2010). This pre-LC complex seems to serve as a carrier of GINS to the existing pre-RC–Sld3–Cdc45 complex through the interaction between Dpb11 in the pre-LC and phosphorylated Sld3 on origins and thereby to provide the preconditions for further activating CMG helicase (Fig. 1A). Another factor, Mcm10, associates with origins and functions later, and this association is required for CMG to move from origins (van Deursen *et al*. 2012; Kanke *et al*. 2012; Watase *et al*. 2012).

In metazoa, RecQ4, Treslin/Ticrr and TopBP1 are counterparts of Sld2, Sld3 and Dpb11, respectively (Table 1). These factors function as described in budding yeast with some differences. RecQ4 associates with chromatin in a TopBP1- and pre-RC-dependent but CDK-independent manner (Sangrithi *et al*. 2005; Matsuno *et al*. 2006). RecQ4 is not required for the association of GINS with chromatin, indicating that it functions at the later stage in the initiation, unlike Sld2 in yeast. Although Treslin/Ticrr and TopBP1 associate with chromatin independently, they bind in a CDK-catalyzed phosphorylation-dependent manner (Kumagai *et al*. 2010). Treslin/Ticrr also binds to Cdc45 and is required for the chromatin association of Cdc45 (Kumagai *et al*. 2010; Sanchez-Pulido *et al*. 2010), similar to yeast Sld3. These results suggest that similar mechanisms occur at the initiation step of DNA replication even in the metazoan activation of DNA helicase.

**Figure 3:**
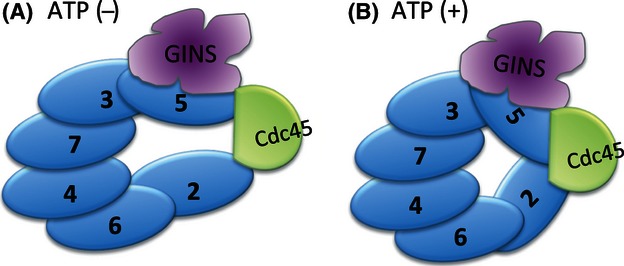
Conformational change of CMG. The CMG complex changes its conformation dynamically dependent on the presence of ATP. (A) In the absence of ATP, Mcm2 and Mcm5 disconnect and generate a big channel. (B) The binding of ATP leads to Mcm2 and Mcm5 closing the Mcm2–7 hexameric ring in assistance with Cdc45 and GINS.

Although we do not know whether the accumulation of replication proteins at origins leads directly to the activation of helicases, there is a strong possibility that such stepwise regulations trigger a series of dynamic changes in the interactions between proteins at origins because of conformational alterations arising from phosphorylation, including the active CMG formation. For example, the helicase hexameric ring should require a ring open/closure procedure or a ring assembly procedure: the helicases must encircle one strand of DNA for unwinding because its single-strand-binding sites are located within the central channel of the hexamers (Davey & O'Donnell 2003). A recent comparison analysis of the structures of MCM2–7 and CMG complex using single-particle electron microscopy provides a more direct and clearer explanation of eukaryotic helicase behavior in replication initiation (Costa *et al*. 2011). Namely, *Drosophila melanogaster* MCM2–7 adopts two conformations: a notched planar form and a lock–washer-shaped spiral state in which the ring breaks between Mcm2 and Mcm5. This gap provides a gate for entry of the DNA strand. With the help of ATP (based on ADP-BeF3 data), MCM2–7 seals the gap and changes the configuration to the notched state, but only for a small percentage. This suggests more factors are involved in the sealing and activation process. Subsequently, Cdc45 and GINS together seal off the open ring with a discontinuity between Mcm2 and Mcm5 of MCM hexamer, thereby reinforcing a more planar configuration with a large interior channel (Fig. 3). This complex, however, still has a discontinuity between Mcm2 and Mcm5 in their C-terminal AAA^+^ domains. Upon ATP binding, the CMG complex closes completely the Mcm2–Mcm5 gate to tighten the MCM hexameric ring (Costa *et al*. 2011).

Why do eukaryotes need complicated steps to activate helicase at the initiation of DNA replication? There are several explanations. First, these steps may regulate replication of local chromosomal DNA. Huge chromosomal DNA spreads throughout the nucleus and origins fire at different times during the S phase. As origins bind to ORC ubiquitously, helicase loading and its activation are regulated in the S phase. In prokaryotes, the level of ATP (ATP–DnaA) affects the association between the initiator protein and origin. However, it is not suitable for the regulation of the local initiation of chromosomal DNA in the eukaryotic multiple-origin system because of the free diffusion of intracellular ATP. Protein phosphorylation, instead, may occur locally because protein kinase can be localized by its affinity to chromatin proteins. Thus, phosphorylation-dependent reactions may regulate helicase loading and subsequent origin firing on chromosomes locally. Second, eukaryotes regulate the initiation of chromosomal DNA replication strictly to keep genome integrity in the cell cycle. The pre-RC is formed from late M to G1 phase in the absence of high CDK activity. Subsequent activation of CDK promotes formation of the CMG complex and inhibits formation of the pre-RC (Diffley 1996). This mechanism ensures origin firing once per cell cycle. In prokaryotes, binding of the initiator proteins to origins itself provokes unwinding of origin DNA. If it occurs in multi-origin eukaryotic cells, total length of single-stranded region unwound at origins is long enough for cells to be damaged and to evoke the cell cycle checkpoint. However, ORC binding alone does not unwind origin DNA in eukaryotic cells, saving the cells from damage and perturbation of the cell cycle. Third, assembly of multiple proteins at replication origins to form active helicase may be suitable for multiple regulations that sense many signals, not only environmental nutrients but also cell–cell interactions etc. Thus, the system may have helped in the evolution of eukaryotic cells to multicellular organisms. Fourth, bidirectional replication forks are established at the initiation of DNA replication. Although DnaB helicase loading to *oriC* has been studied extensively in *E. coli*, the molecular mechanism responsible for the formation of bidirectional forks is not well understood. In eukaryotic cells, head-to-head loading of MCM2–7 helicase would ensure establishment of the bidirectional forks, and the activation of MCM2–7 may secure these forks. Although the underlying mechanism remains unknown, it may be inferred that head-to-head–loaded MCM2–7s are activated simultaneously by additional step(s). Further study will uncover the mechanism responsible for this simultaneous activation.

## Conclusions

ATP binding and hydrolysis seem to be the common processes used to regulate bacterial replicative helicase loading and activation. Eukaryotes use finer regulatory systems, including control of stepwise DDK and CDK activities, in addition to ATP binding and hydrolysis. Compared with bacterial helicase, in which unwound origin DNA is loaded directly and is followed immediately by its activation, the eukaryotic replicative helicases take a detour. The catalytic unit MCM2–7 first loads onto double-stranded DNA without helicase activity and then switches to tracking along single-stranded DNA, which coincides with the active CMG formation. Bacterial helicases including *E. coli* DnaB and *B. subtilis* DnaC assemble into homohexameric rings, each with a central channel that is sufficient for inducing their activities. These helicases unwind DNA progressively in the 5′–3′ direction. Interestingly, MCM2–7 heterohexamer serves only as a catalytic core and requires Cdc45 and GINS to exert its full activity. The activated helicase CMG exerts unwinding activity in the 3′–5′ direction. These differences underline the diversity in the detailed mechanisms and strategies that various species have adapted in the processes that duplicate their genomes. Recent advances in research have identified more variations in functional orthologues in sequences or structures between species. At present, we do not know much about DNA replication and replication factors in plant cells. Further investigation of DNA replication in various organisms will identify the ultimate and integral similarity.
